# The interplay between macronutrients and sleep: focus on circadian and homeostatic processes

**DOI:** 10.3389/fnut.2023.1166699

**Published:** 2023-08-23

**Authors:** Elena Gangitano, Matthew Baxter, Maria Voronkov, Andrea Lenzi, Lucio Gnessi, David Ray

**Affiliations:** ^1^Oxford Centre for Diabetes, Endocrinology and Metabolism, University of Oxford, Oxford, United Kingdom; ^2^Department of Experimental Medicine, Sapienza University of Rome, Rome, Italy; ^3^National Institute for Health Research Oxford Biomedical Research Centre, John Radcliffe Hospital, Oxford, United Kingdom

**Keywords:** sleep, macronutrients, circadian process, homeostatic process, process C, process S, clock, protein

## Abstract

Sleep disturbances are an emerging risk factor for metabolic diseases, for which the burden is particularly worrying worldwide. The importance of sleep for metabolic health is being increasingly recognized, and not only the amount of sleep plays an important role, but also its quality. In this review, we studied the evidence in the literature on macronutrients and their influence on sleep, focusing on the mechanisms that may lay behind this interaction. In particular, we focused on the effects of macronutrients on circadian and homeostatic processes of sleep in preclinical models, and reviewed the evidence of clinical studies in humans. Given the importance of sleep for health, and the role of circadian biology in healthy sleep, it is important to understand how macronutrients regulate circadian clocks and sleep homeostasis.

## 1. Introduction

### 1.1. Sleep disturbances and metabolic disturbances: an inseparable duo

The association between obesity and sleep has been increasingly studied over the last years. The duration of sleep has been put in relation to metabolic disturbances in many clinical studies. Metabolism and sleep mutually influence each other, in a complex relationship. Lack of sleep is associated to a higher body mass index (1–5) and to central obesity (4, 6–10). The recommended amount of sleep, according to the consensus statement of the American Academy of Sleep Medicine and Sleep Research Society, is at least 7 h per night on a regular basis (11).

Central obesity, which is characterized by visceral fat deposition, is a metabolic feature associated with a higher cardiovascular risk. Lack of sleep is associated to glucose metabolism impairment and type 2 diabetes (12, 13). A sleep duration longer of just 1 h per night is associated with a lower prevalence of obesity and type 2 diabetes (14, 15). Similarly, a reduction of just 1 h of sleep per night is associated with weight gain (1).

It is known that sleep restriction can stimulate appetite (16–18) and seeking for high calorie food (16, 19, 20). This is particularly evident in the conditions in which the sleep–wake cycle is disturbed, as in shift work (21). In fact, it may lead to an increased preference for calorie-dense foods (22), reduction in energy expenditure (23) and alterations in appetite-controlling hormones (23). On the other hand, food acts as an external regulator of the circadian rhythm of the individual, of which sleep is a major manifestation (24).

There is some evidence that the relationship between BMI and sleep duration is U-shaped, so that long or short sleep durations are both associated to increased adiposity (15, 25). Similarly, the relationship between sleep duration and impaired glucose tolerance or diabetes is U-shaped (12, 13), so that we may suppose that metabolic disturbances, whatever they are, are similarly interconnected with sleep duration, but that sleep duration cannot be considered as a linear variable. The variability in sleep duration can be itself considered a risk factor for metabolic alterations (26), diabetes (14) and adiposity parameters (4, 27, 28) at any age. Also sleep quality has its own importance. Given the fact that sleep is composed by quantitative features that are objectively measurable, as sleep latency and sleep duration, and some aspects that are mainly subjective and impossible to measure objectively, as depth and restfulness (29), sleep quality is a parameter that is difficult to define in its entirety. In fact, it is determined by the combination of many factors, as sleep duration, latency, fragmentation, and the perception of restorative sleep, which are strictly connected to the presence of daytime dysfunction. A higher degree of sleep fragmentation, calculated considering movement and immobile epochs with the actigraphy, is related to a shorter total sleep time and associated with obesity (25), and a reduced sleep efficiency is associated to central obesity in women (30).

On the other hand, obesity is a risk factor for developing sleep disturbances. Obesity can increase the risk of developing obstructive sleep apnoea (31), and the psychological and environmental implications of obesity can affect sleep quality, so that sleep disturbances and obesity sit in a vicious circle in which one worsen the other (32).

The mechanisms of the association between lack of sleep and metabolic disturbances are not clarified yet. Metabolic disturbances, included diabetes and non-alcoholic liver disease, are strictly connected among each other, and they similarly interact with lack of sleep. Metabolic disturbances are often associated to hormonal imbalances, which may play a role in their development, in a complex relationship of mutual influence (33–36). Some hormones may be involved in the association between metabolic disturbances and reduced sleep. The higher levels of cortisol due to the condition of chronic stress related to sleep deprivation may explain this relationship, through the increased levels of insulin resistance and inflammation (37). Moreover, the appetite regulation hormones are altered, and lead to an increased energy intake. Lack of sleep is associated to higher levels of pro-inflammatory cytokines and altered sympathetic activity, which lead not only to weight gain itself, but also to the development of a higher cardiovascular risk through the induction of endothelial dysfunction (32, 38).

### 1.2. Sleep structure and functioning

#### 1.2.1. Sleep architecture

Sleep is a physiological element which is particularly important for health, and specifically, for metabolic and cardiovascular health. It is composed by a rapid-eye movement phase (REM) and non-REM phases. The non-REM phases include the phases N1 and N2, which are the “light sleep” phases, and N3 that is the “slow wave sleep” phase (SWS), that is a deep sleep phase. Both REM and slow-waves sleep contribute to the restorative function of sleep. Sleep stages succeed one after the other in cycles that last from 90 to 110 min. Each stage lasts differently: N1 last from 1 to 5 min, N2 from 10 to 60 min and SWS from 20 to 40 min, while REM phase lasts from 10 to 60 min. These lengths vary during the sleep, and REM length increases along the night, and the non-REM phase gets shorter. The majority of sleep is spent in N2 phase, and non-REM phase lasts about 75% of sleep. During a night, normally there are 4 o 5 sleep cycles, with the progression across sleep stages as N1, N2, N3, N2, REM (39).

#### 1.2.2. Sleep components: the homeostatic and circadian processes

**Figure 1:**
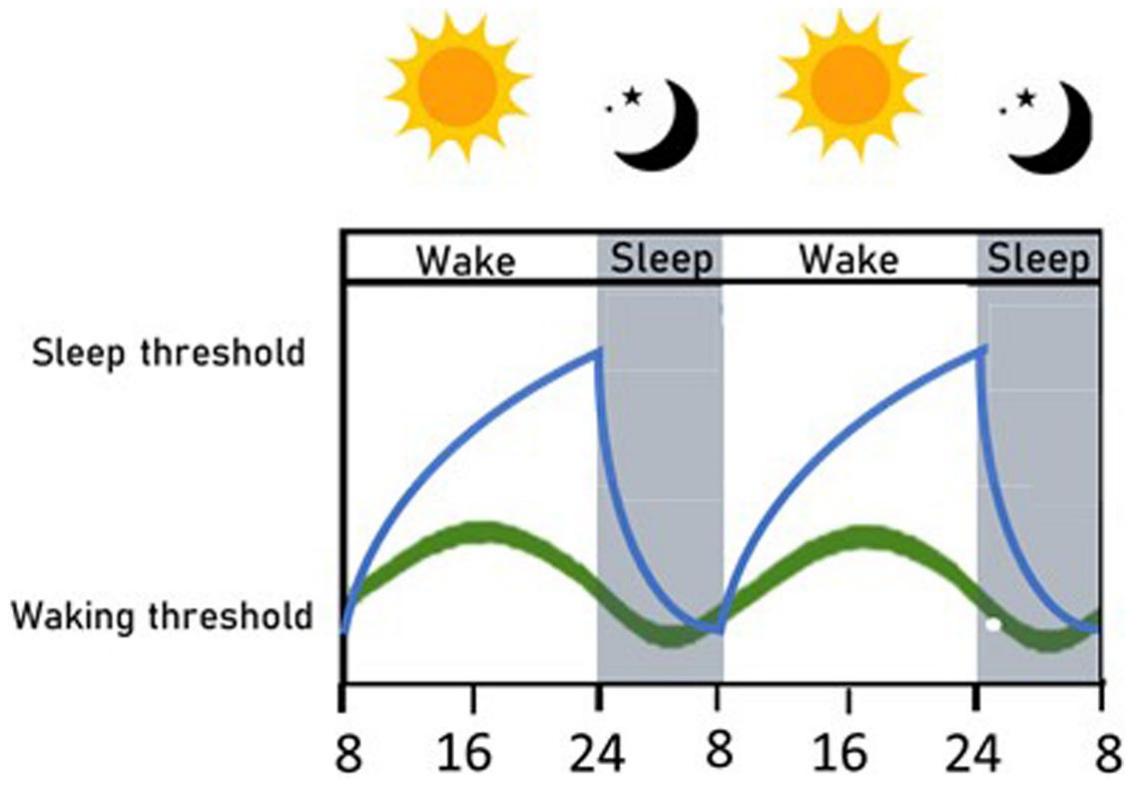
Circadian and homeostatic sleep processes. A simplified graphical representation of the two sleep processes, within a 2 days-time, in physiological conditions. The circadian process (green line) helps set the timing of sleep, and its fluctuations are regulated by the alternance of light and dark. The homeostatic process (blue line) is related to sleep debt, which accumulates while awake, and once the sleep threshold is reached, promotes sleep; then sleep debt decreases and awakening occurs when the waking threshold is reached. The two processes are interconnected and mutually interact, so that sleep is harmoniously regulated (40).

Four decades ago, Borbély proposed the two-process model of sleep (40): a conceptual model that has shaped the field of sleep research and is still the primary model used to describe sleep processes. This model describes the continuous interaction of two processes, termed process S and C, that are responsible for driving sleep and wake timing (see Figure 1). Process S, or the homeostatic process, represents sleep debt, which builds up during the wake phase and decreases during sleep. When it reaches its lowest value at the end of sleep, it triggers the body to awaken, and when it reaches its highest value after a long period of being awake, it triggers sleep. Meanwhile, process C represents the circadian process. This is controlled by the master circadian regulator, which resides within the Suprachiasmatic Nuclei (SCN). Process C oscillates across a day-night cycle, as entrained by external light cues, and controls the timing of sleep and arousal. The two components are interconnected and mutually influence each other to determine sleep propensity (40) in a complex relationship, not yet completely elucidated, that influences some characteristics of sleep, as length and depth; in fact, circadian amplitude may be reduced when sleep pressure increases (41). In physiological conditions, the two processes are harmoniously concordant, so that sleep onset is favored by both processes during the night, when it is dark, and sleep debt has accumulated during the day. Clock genes, the fundamental bricks of the circadian process, are also implicated in sleep homeostasis; the expression of some clock genes changes with wake and sleep phases and with sleep deprivation, and conversely sleep homeostasis is altered in many clock gene mutants (42).

For the purpose of this review, we will focus on how macronutrients influence the sleep components.

## 2. Circadian process and macronutrients in pre-clinical models

### 2.1. The circadian clock and the role of dietary nutrients on its entrainment and function

The two-process model of sleep regulation describes the interaction of a homeostatic process S dependent on time spent asleep/awake, and process C which is controlled by the central circadian pacemaker (43). The ‘master’ circadian clock resides within the Suprachiasmatic Nuclei (SCN) which is located in the hypothalamus. The SCN receives direct light input from the retinal ganglia in order to ‘tell time’. In practice, this means that the master clock of the SCN is entrained by exposure to external light cues, and can shift by approximately 1 h per day in humans and mice. The period of the circadian clock in the SCN (the amount of time it takes to complete one cycle) is approximately 24 h in order to match the rotation of the Earth about its axis. The exact amount of time for one oscillation in the absence of external stimulation is known as the ‘free-running period’ and varies on average between species and individuals, and may be slightly more or less than 24 h. The core circadian clock controls 24-h cycles not only of sleep/wake, but also other physiological parameters, such as core body temperature and blood pressure, as well as secretion of hormones such as melatonin, cortisol, and prolactin.

At a molecular level, the circadian clock is composed of a transcription-translation feedback loop. The positive arm of this loop includes the proteins BMAL1 and CLOCK, which activate the transcription of the inhibitory PERIOD and CRYPTOCHROME (PER1-3 and CRY1&2) proteins. The oscillations are stabilized by an auxiliary loop including REVERB and ROR proteins. This molecular clock operates not only in the SCN but also in peripheral tissues and cell types, with signals from the central SCN pacemaker synchronizing the peripheral clocks (44). Peripheral clocks are influenced by feeding and fasting states and are kept synchronized with the central clock. In this physiological situation, light and dark cycles succeed harmoniously, in accordance with feeding and fasting status. However, peripheral clocks can become decoupled from the central clock, due to stimuli including, for instance, mis-timed feeding, i.e., eating out of synchrony with the biological clock, during normal physiological rest phase (45). This decoupling has been linked to the pathogenesis of a wide variety of diseases, including metabolic disorders. Both central and peripheral circadian clocks are extremely important for regulating the metabolism of macronutrients and maintaining energy homeostasis (46).

**Figure 2:**
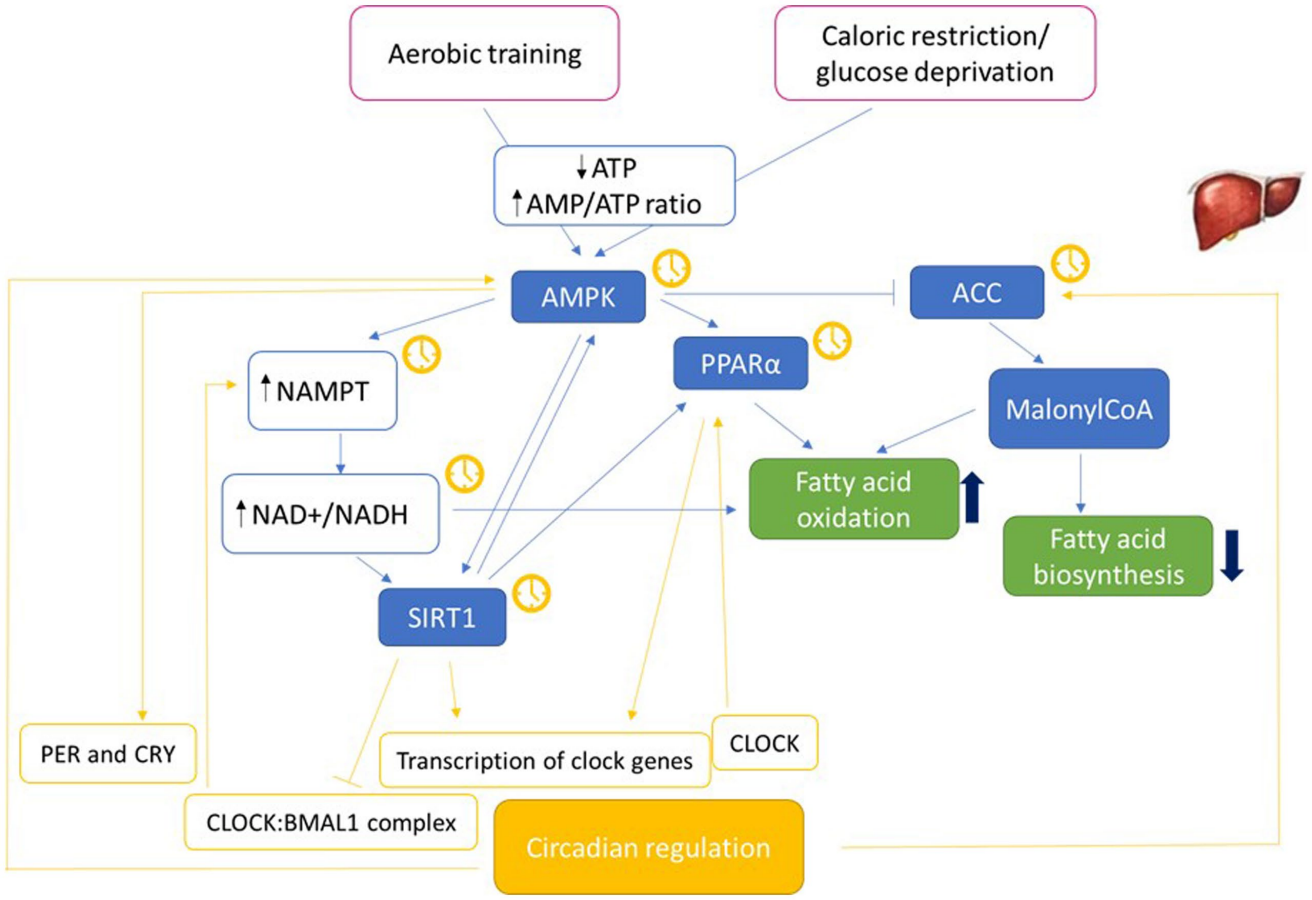
Molecular pathways possibly involving metabolic functions and circadian rhythm. Molecular pathways that may represent the connection between metabolism and circadian machinery (46, 71, 175–184). NAD+ acts as a cofactor for fatty acid oxidation and SIRT1 activity. The yellow clock indicates the molecules whose expression is circadian. Adenosine monophosphate-activated protein kinase (AMPK); Nicotinamide phosphoribosyltransferase (NAMPT); nicotinamide adenine dinucleotide (NAD+/NADH); Sirtuin 1 (SIRT1); Peroxisome proliferator-activated receptors (PPAR); Acetyl-CoA carboxylase (ACC).

Metabolites from feeding play a role in regulating cellular rhythmicity, therefore diet composition play a role in influencing circadian clock activity and reprogram the clock in mice (47). The mechanisms through which nutrients impact circadian metabolome, and consequently biological rhythm, have not yet been fully elucidated and results are not completely concordant (see Figure 2). Research in animal models showed that different diets have a different impact on circadian phase.

**Figure 3:**
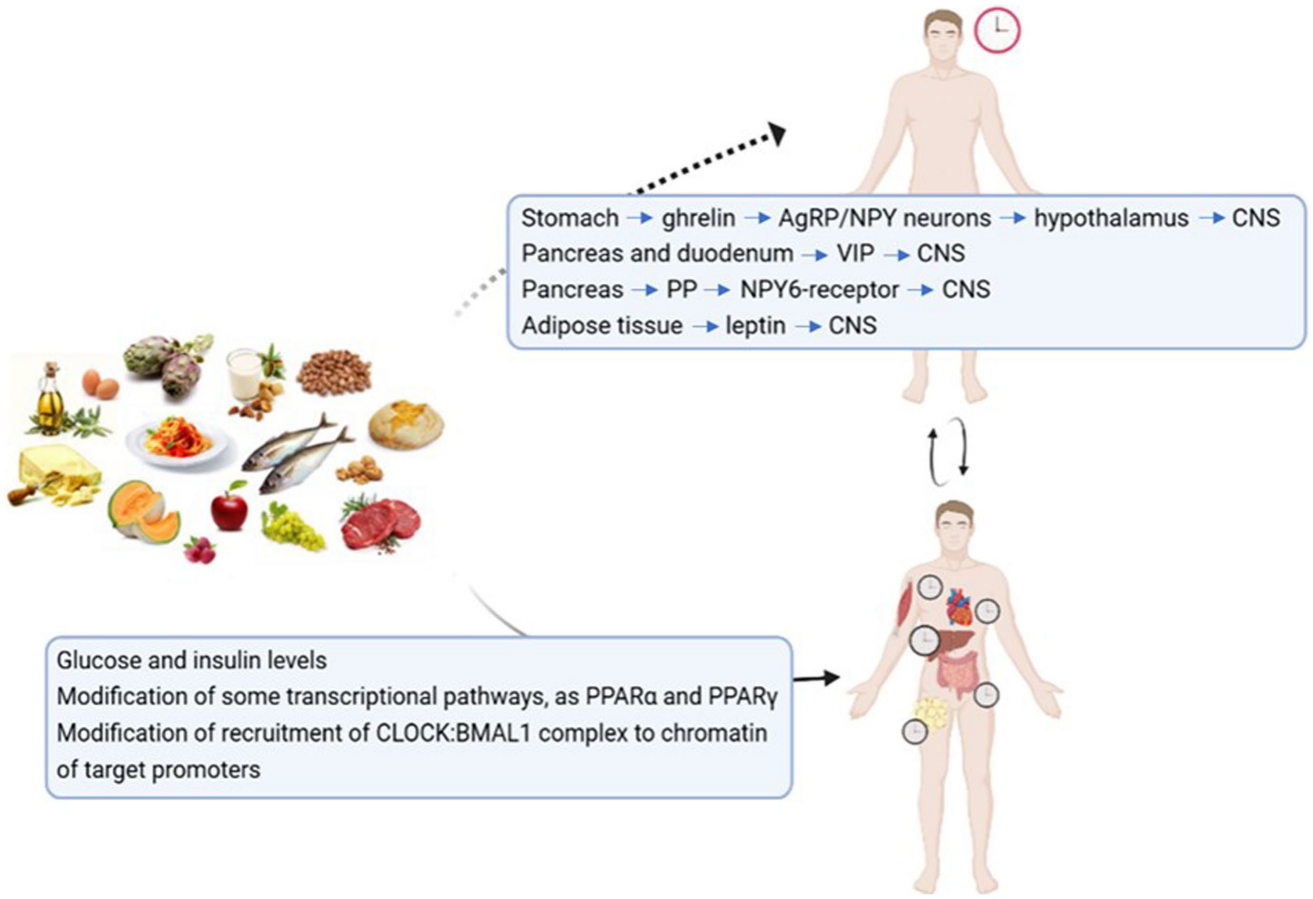
How food may affect the central and the peripheral clocks. Possible mechanisms through which nutrients may act on the central and the peripheral clocks (47–49, 185–187). Central Nervous System (CNS); Vasoactive intestinal Peptide (VIP); Pancreatic Polypeptide (PP); Peroxisome-Proliferator Activated Receptor (PPAR).

Nutrients act mainly on the peripheral clocks, but some preliminary evidence suggest that food is able to directly interact with the central clock (48) (see Figure 3). During fasting conditions, the production of ghrelin from the stomach activated AgRP/NPY neurons, that project to the arcuate nucleus of the hypothalamus, and then connect to the CNS. Also the intergeniculate leaflet receives information form the periphery and is able to project to the CNS. Another mechanism could involve the pancreas, that in response to food intake produces pancreatic polypeptide that activate NPY6-receptor (Npy6r) that is coexpressed with vasoactive intestinal polypeptide (VIP) in the CNS of mice, and synchronizes CNS neurons. Leptin may be involved as well, since it has been observed that *in vitro* is able to forward shift the peak time of CNS and has a role in modulating the CNS response to light. Mendoza et al. (49) administered HF diet to mice and observed that the induction of the early gene c-fos by light in CNS was reduced, maybe through VIP signaling, so that it is possible hypothesize that a HF diet modifies circadian synchronization to light. *c-fos* is a regulatory protein found within the SCN, considered as a molecular marker of SCN resetting. The production of ghrelin, VIP and leptin is differently influenced by different macronutrients, as for example glucose markedly suppresses ghrelin concentration, medium-chain triglycerides reduce it less strongly, while amino acids determine a rise in ghrelin levels (50), therefore we may hypothesize that different macronutrients, through gastrointestinal hormones levels, differentially impact central clocks.

### 2.2. Impact of macronutrients on circadian process in pre-clinical models

#### 2.2.1. Dietary fat effects on central and peripheral clocks

Experiments in mice have served as the main model for examination of the mechanisms of circadian clock function, and it is important to note that laboratory mice are naturally nocturnally active whereas humans are naturally diurnally active, i.e., the circadian active phase of mice is at night and the active phase of humans is during the day. Importantly, studies in mice have shown that the circadian phase of the SCN is not affected by restricting food intake to the rest phase, whereas the phase of peripheral organs, especially the liver, can be (51, 52). This observation indicates that circadian clocks in different tissues can become uncoupled, and this uncoupling, or internal desynchrony, has been associated with multiple human diseases (53). Examination of this phenomenon in mice using jet lag studies and genetic knockouts has shown a direct link between the circadian clock genes and metabolic disease phenotypes (54). Furthermore, disruption of the circadian clock by high-fat diet (HFD) and constant light have independent and additive effects on weight gain in mice, indicating that disrupted circadian rhythms potentiate the detrimental effects of high-fat diet (55).

The SCN receives metabolic feedback from peripheral organs, as evidenced by the observation that the free-running period of mice is altered by exposure to high fat diet (49). The induction of the gene c-fos by light was reduced in the SCN of mice fed high-fat diet, and this effect may be mediated by vasoactive intestinal peptide (VIP) and neuropeptide Y (NPY) (49). Metabolic entrainment of the SCN may also occur through the adipokine leptin, which can cause dose-dependent phase-shift of SCN tissue *in vitro* (56). Administration of exogenous leptin did not shift the behavioral circadian rhythm of mice *in vivo*, but did potentiate the effect of light-pulse induced phase-shift through induction of the clock genes Per1 and Per2 (57). Other studies have found that neurons from the arcuate nucleus and intergeniculate leaflet, which are sensitive to the appetite related hormone ghrelin, transmit signals to the SCN, and thus NPY and GABA may also provide metabolic feedback to the central circadian clock (58).

**Figure 4:**
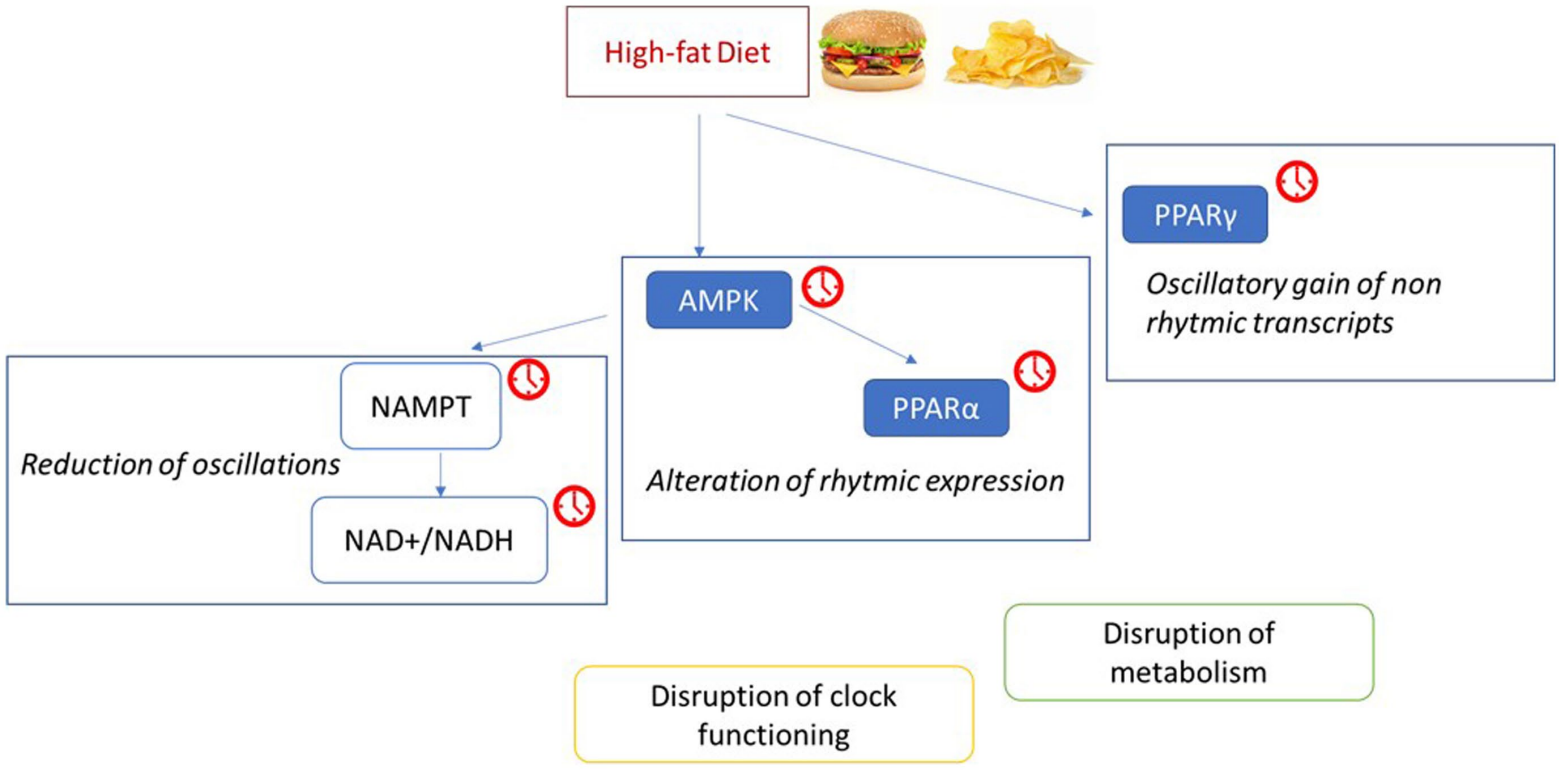
The high-fat diet induces a reprogramming of metabolic pathways and circadian rhythm. Possible mechanisms through which a high fat diet disrupts clock function and metabolism (47, 62, 188), inducing an altered or a phase shifted recruitment to chromatin of target genes by CLOCK:BMAL1. The red clocks indicate the molecules whose expression during the day is altered by the consumption of a high-fat diet. Adenosine monophosphate-activated protein kinase (AMPK); Nicotinamide phosphoribosyltransferase (NAMPT); nicotinamide adenine dinucleotide (NAD+/NADH); Peroxisome proliferator-activated receptors (PPAR).

While dietary fat has been shown to exert some feedback on the central pacemaker, a much greater effect is observed on clocks in peripheral tissues. Clocks in peripheral metabolic tissues, such as adipose tissue, are hypothesized to regulate energy partition in response to regular daily feeding patterns to help maintain energy homeostasis. Therefore, timing of macronutrient intake may be of high importance, and indeed mice fed HFD at the end of the active phase exhibit greater adiposity than those fed HFD at the beginning of the active period. This phenomenon may be an effect of disturbed temporal regulation of β-oxidation (59). Other studies have shown that restriction of HFD to the rest phase leads to significantly higher weight gain than restriction of HFD to the active phase (60). Furthermore, restriction of HFD to active phase when compared to *ad libitum* HFD reduces body weight gain, increases glucose tolerance, and prevents the development of metabolic liver disease. This may be related to a restored efficiency in the pathways function of CREB, mTOR and AMPK, and improved oscillation of the clocks and their target genes (61). HFD significantly alters the rhythmic expression of clock genes in the liver, as well as clock output genes (62, 63) (see Figure 4). This includes, Pparα and Ampk which promote β-oxidation as part of the adiponectin signaling pathway, and disruption of their normal rhythmic expression may lead to impaired liver lipid metabolism. HFD determines the loss of rhythmic metabolite expression, such as NAD+, that parallels the dampened cyclic transcription of Nampt. These modifications in the rhythm of gene expression induced by the HFD may alter the circadian clock by disrupting, through a phase-shift or a reduction, of the recruitment of BMAL1: CLOCK to its target promoters on chromatin (47). At the same time, HFD determines oscillatory gain of normally non-rhythmic transcripts with known or predicted methyltransferase activity, such as *Ehmt*, *Trmt2b*, *Whsc1*, and *Dph5*. The transcriptional reprogramming by a HFD is related to the oscillation and chromatin recruitment of PPARγ, whose expression is induced by a HFD (47).

#### 2.2.2. Glucose effects on central and peripheral clocks

Glucose homeostasis is subject to circadian control, as surgical lesion of the SCN in rats abolishes the 24-h rhythm of basal blood glucose and insulin (64). But glucose may also exert a reciprocal effect on the circadian clock, and there is evidence of regulation in the SCN. Parenteral administration of glucose caused a phase-shift in SCN Per2 mRNA expression in rats (65). Furthermore, *in vivo* administration of insulin to induce hypoglycaemia in mice decreased the magnitude of light-pulse induced phase shifts, indicating that brain glucose levels are important for circadian entrainment (66).

*In vitro*, glucose has been shown to downregulate Per1 and Per2 gene expression in rat fibroblasts, possibly mediated by transforming growth factor β-inducible early gene 1 (Tieg1 (67, 68);. Conversely, insulin upregulates Per1 and Per2 gene expression in rat fibroblasts (69). Mice treated *in vivo* with streptozotocin to induce non-obese insulin resistance, showed alterations in hepatic expression of Bmal1, Per2, and Cry1, which were reversible by PPARα agonist pioglitazone (70). This might suggest that PPARα mediates the feedback of glucose homeostasis on to the core circadian clock. Indeed, multiple studies have shown that PPARα agonists change the rhythmic expression of core clock genes in multiple mouse tissues (71–74). Interestingly, PPARα agonist bezafibrate caused behavioral phase-advance in SCN-lesioned mice, indicating that the peripheral PPARα may be able to exert circadian control independently of the SCN master clock (75). Other potential mediators include the kinases Mapk and Gsk3, both of which are regulated by insulin signaling. Mapk can downregulate Bmal1, whereas GSK3 phosphorylates and thus degrades CRY2 (76, 77). The exact role of GSK3 in circadian entrainment is poorly understood, with opposing observations in different systems. Pharmacological inhibition and siRNA knockdown of GSK3 caused period lengthening in cultured mouse SCN and fibroblasts (78, 79). Whereas in cultured human U2OS cells, inhibitors and knockdown of GSK3 lead to period shortening (80). Another glucose sensing kinase, Ampk, can also phosphorylate and destabilize CRY1 which may add another layer of complexity to the feedback mechanisms between glucose and the circadian clock (81).

#### 2.2.3. Dietary protein effects on central and peripheral clocks

Relatively less research has been conducted on the role of dietary protein in circadian clock entrainment. Most of the few research articles examining this have used “Essence of Chicken” (EC), which is a mixture of water-soluble substances derived from cooking whole chicken. Whilst EC contains many proteins and peptides, it will also contain many other water-soluble factors, and it is important to note that standardization is likely to be difficult to achieve. However, supplementary dietary protein has been shown to enhance the circadian oscillation of glucocorticoid secretion in mice (82), indicating a potential role for certain amino acids. In mice exposed to 12 weeks of light-induced circadian disruption, dietary EC supplementation was reported to decrease the time to reset clock genes in the pineal gland (83). Further examination of this model, which induces decoupling of hepatic clock genes (Per1, Cry1, Bmal1) and key metabolic regulators (mTOR, AMPK), and leads to liver injury, showed that dietary EC reduced markers of liver inflammation and improved synchronicity of clock genes in liver (84). It should be stressed that the role of specific proteins or amino acids cannot be delineated from other non-protein constituents of the dietary supplement in these studies. However, another study, showed that protein-only diet and/or amino acid (cysteine) administration, can elicit entrainment of liver clock via glucagon secretion and production of IGF-1 (85). Furthermore, an *in vitro* study demonstrated that exogenous methionine and arginine can alter the phosphorylation of mTOR and the expression of circadian clock proteins, offering a plausible mechanism for the *in vivo* observations mentioned above (86). Lastly, it is noteworthy that protein ingestion has differential effects on intestinal microbiota in different circadian phases, and intestinal microbiota may play a role in regulating peripheral circadian clocks (87).

## 3. Homeostatic process and macronutrients in pre-clinical models

### 3.1. Molecular mechanisms of process S

Unlike the rhythmic circadian process C, homeostatic process S is cumulative – with sleep debt accumulating to its maximal value while awake, and decreasing exponentially during sleep. Higher levels of sleep debt result in an increased intensity in sleep, affecting the amount of stimuli required to awaken (88–91). While the exact molecular mechanisms involved in process S have yet to be elucidated, current thinking in the field centers around the hypothesis that process S is driven by the build-up of neuro-modulating substances in the brain, as originally proposed by Feinberg et al. and Borbély et al. (92, 93).

The primary marker used to measure process S is the presence and amplitude of slow wave activity (SWA) as recorded by electroencephalogram (EEG). This is typically considered to be in the range of 0.5–4.0 Hz (43). SWA correlates almost perfectly with sleep debt: slowly increasing over the course of the waking period, peaking during early sleep, and declining over the course of sleep. SWA is caused by the rhythmic firing of cortical neurons, particularly during non-rapid eye movement (NREM) sleep, in which neurons synchronously switch between a bursting and resting phase. As sleep need decreases, synchrony is diminished and the SWA pattern is lost (94).

One of the proposed neuro-modulating substances responsible for driving homeostatic sleep debt is adenosine (95). This was first proposed by Benington and Heller, where they postulated that the role of sleep was to replenish depleted glycogen stores, and that SWA was caused by the release of extracellular adenosine by glycogen-depleted astrocytes (96). While this model has proved to be a very simplistic view of sleep (97), the proposed links to adenosine secretion have been verified by further studies (98). Adenosine is bound via the adenosine A1/A2 receptor in various parts of the brain and, interestingly, can trigger certain pathways typically involved in light-sensing via cAMP-CREB and ERK signaling (99). This may indicate a potential cross-over point for process C and process S. However, knockdown of adenosine A1 receptor has been described to disrupt SWA, a readout of homeostatic sleep drive, but not total sleep duration, which would typically be controlled by process C (100).

More recently, a phosphorylation-centric model of sleep debt has been proposed in which synaptic protein phosphorylation may be the neuro-modulator responsible for dictating sleep pressure (101). This is evidenced by the discovery of sleep-inducing kinases, including calmodulin-dependent protein kinase (CaMK) II (102), salt-inducible kinases (SIK)3 (103), and extracellular signal-regulated kinases (ERKs) (104), which increase in activity over the wake period and show elevated function during sleep deprivation. In addition, the synaptic phospho-proteome shows a peak cluster of phosphorylation events at sleep–wake transition periods that is independent from circadian control (105), as demonstrated by sleep deprivation studies. It is therefore plausible that phosphorylation events are a key molecular mechanism underpinning process S. Moreover, these sleep-inducing kinases are implicated in metabolic pathways as well. CaMKII is activated by exercise and influence lipid metabolism (106), SIK3 belongs to the AMP-activated protein kinase (AMPK) family, regulates cholesterol and bile acid metabolism and influence lipid metabolism (107, 108), while the MAPK/ERK pathway recently emerged as a key regulator of the Warburg effect, an aerobic glycolysis metabolic reprogramming occurring in cancer cell proliferation and some physiological processes (109).

There is a growing body of evidence that different macronutrients can affect various sleep parameters, including sleep intensity and sleep pressure. The next section of this review will focus on the impact of different macronutrient groups on the homeostatic component of sleep in pre-clinical models.

Circadian rhythm is tightly controlled and fundamental for the health of the living organism. In fact, the regulation of circadian rhythm is redundant, given the role of neuronal PAS domain protein 2 (NPAS2), which can functionally substitute CLOCK in the central clock in mice (110). NPAS2 is a conserved gene whose transcript can dimerize with BMAL1 and activate the transcription of genes related to circadian rhythm, as PER1/2/3 and CRY1/2 (111). Sleep deprivation reduces the binding of CLOCK, NPAS2 and BMAL1 to the promoters of target circadian genes. In particular, NPAS2 is able to induce sleep when the sleep need increase, even when it is not the time to sleep, therefore playing a major role in sleep homeostasis, probably acting at the thalamus and cortex, where it is more expressed (112). In addition, NPAS2 has also a metabolic role, since it boosts the Warburg effect in hepatocellular carcinoma cells through increasing the expression of glycolytic genes (111).

### 3.2. Impact of macronutrients on homeostatic component of sleep in pre-clinical models

#### 3.2.1. Carbohydrates and homeostatic sleep

Carbohydrates are one of the best described macronutrients that affect sleep. In particular, it has been proposed that high levels of glucose result in the uptake of tryptophan (Trp) in the brain (113). Following ingestion of a high carbohydrate meal, insulin influences tryptophan supply to the brain by both promoting the binding of tryptophan to albumin, and at the same time reducing the levels of several other amino acid species that would usually compete with tryptophan for transport proteins into the brain (114). Tryptophan is a precursor for serotonin and ultimately melatonin, which is the primary sleep-promoting hormone, suggesting that carbohydrate intake could promote sleep pressure.

Unfortunately, few studies have been performed looking at the role of carbohydrates during sleep in rodent models. However, one paper found that glucose activates sleep-promoting neurons in the ventrolateral preoptic nucleus in mice (115), which are essential for the initiation and maintenance of SWA. The authors found that physiological levels of glucose led to the selective excitation of sleep-promoting neurons, which is normally gated by K_ATP_ channels. Induction of SWA is a marker of increased sleep pressure, and therefore suggests that glucose intake promotes sleep in mice. SWA has also been shown to regulate glycolytic metabolism directly (116). Measurement of lactate levels in real time reveal that lactate builds up during wakefulness and depletes during SWA sleep proportionally to the amplitude of SWA oscillations. Therefore, this suggests that SWA reduces the glycolytic rate of the cerebral cortex, and thus glucose metabolism is an essential part of homeostatic sleep.

The sleep modulating role of carbohydrates has been better studied in Drosophila, where response to sugar intake appears to be dose dependent. Sleep pathways between Drosophila and mammals are generally considered to be very highly conserved, with both exhibiting the two-process model described by Borbély et al. (43), therefore conclusions from Drosophila are likely to be relevant to human biology. Starvation in flies results in a dampening of sleep, leading to hyperactivity as they redirect their priorities to locating a new food source (117). This is conserved in mice, with food restriction promoting wakefulness and delayed onset of sleep during daytime (118). This can be rescued by detection of a sweet gustatory stimulus, promoting an induction of sleep and reduction in locomotor activity, such as feeding of low sucrose concentrations (119–121). This gustatory rescue of the starvation effect may be of interest in humans, for maintaining a good sleep in cases of food shortages and unstable environmental conditions (119). Interestingly, meals containing high sucrose concentrations (between 5 and 33%) have a polar effect, instead resulting in increased movement and delayed sleep (122). When maintained on a high sucrose diet over a long time period, flies show a reduction in number of sleep bouts but increased average sleep length compared to low-sucrose fed flies (123). As flies age, their sleep patterns fragment and sleep quality drops. Chronic feeding of high sucrose in aged flies has no effect on reduction of age-induced sleep fragmentation, however a controlled short exposure to a high sucrose feed can rescue this phenotype (124), suppressing sleep fragmentation in aged flies for a short time period, highlighting that the feeding of different macronutrients may be used as a treatment for certain types of sleep disruption. The effect of dietary sugar on sleep behavior is independent of circadian rhythm (123), as it is conserved even in models where circadian rhythm is disrupted, indicating that it primarily acts on the homeostatic sleep process.

Similarly to taste, also smell has a connection with sleep, as the perception of odors is reduced in some sleep stages and, conversely, odors can affect sleep, through the modulations of arousal and sleep latency, duration and quality. Moreover, sleep improves the odor memory (125). Some odors are related to ancestral mechanisms, so that the sleep of newborn infants is ameliorated by the smell of the mother. In rodents, the odor of food stimulates the awakening, which is stronger when the animal is food-deprived (125). The odor itself is able to determine a metabolic response in mice, so that increased or reduced smell ability is associated to modifications in body weight, probably due to modifications of energy expenditure, and not only to the modifications in food intake that may occur (126). Metabolism is strictly connected with aging and life span, therefore the connection between smell, metabolism and sleep may be of particular interest. If these findings are applicable to humans, which express much less olfactory receptor genes, and have a smaller olfactory bulb, is still to be elucidated.

#### 3.2.2. Protein and homeostatic sleep

Consumption of protein has also been described to affect sleep architecture. In particular, addition of 2% yeast protein to the diet of drosophila resulted in increased locomotor behavior, reduced sleep duration, and lowered the arousal threshold required to awaken, indicating shallower sleep (122). However, this effect may depend on the specific ratio of carbohydrate: protein, as the addition of protein to a high sucrose diet instead promotes the consolidation of sleep in flies (123), reverting the sleep disruption normally observed in high sucrose diet fed flies. Therefore, it may be more useful to look at the relative composition of different macronutrients in a meal for therapeutic purposes, rather than study each macronutrient in isolation.

In mice, maternal diet composition plays a significant role on offspring health (127). Offspring born to murine dams fed a restricted protein diet display altered energy expenditure and disrupted sleep architecture compared to normal protein fed controls, despite being fed identical diets after weaning. Interestingly, these changes were independent from circadian machinery, and may indicate that protein consumption during gestation is particularly important for development of homeostatic sleep circuitry.

Tryptophan-rich proteins have a particular role in sleep promotion, as mentioned in the previous section. Consumption of the essential amino acid tryptophan directly facilitates sleep via the generation of melatonin (114). This is dependent upon tryptophan availability in the brain, which is controlled by the ratio of tryptophan to branched chain amino acids in circulation. This is aided by insulin signaling, which actively reduces the availability of branched chain amino acids and therefore promotes the uptake of tryptophan by transport proteins in the brain. However, melatonin appears to control sleep primarily via circadian factors, as it does not seem to alter sleep need as measured by sleep duration, and therefore this is unlikely to affect the homeostatic process (128). Some other authors observed that tryptophan has a direct effect on the homeostatic regulation of sleep. The re-feeding of rats after 4 days of fasting with a diet rich in α-lactalbumin was able to restore the time spent in SWS and wakefulness in just 1 day of refeeding, contrarily to the rats fed with a control-chow with lower amount of lactalbumin, who needed 4 to 6 days of refeeding to restore the alteration in the sleep architecture induced by the fasting (129). Probably, this happened through an increased transport of tryptophan through the brain–blood barrier, as lactalbumin has a higher Trp: large neutral amino acids (LNAA) ratio, and therefore the availability of brain serotonin increased. In fact, tryptophan is the serotonin precursor, through a short metabolic pathway of two enzymes, the tryptophan hydroxylase and the amino acid decarboxylase (130). Serotonin is a neurotransmitter implicated in the regulation of several behavioral and physiological function, as mood, cognition, but also sleep and wakefulness (130), and serves as a precursor for the synthesis of melatonin in the pineal gland during the night.

#### 3.2.3. High fat diet and sleep need

The effects of lipid intake on sleep have primarily been studied in high fat diet obesity models, rather than isocaloric meals containing altered lipid proportions, so it is difficult to separate the effects of dietary fats from that of obesity-related behavioral changes. In mice, high fat diets have been shown to increase sleep pressure, reduce wakefulness, and fragment sleep, as supported by a significant increase in SWA during both wake and rest phases (131, 132). Alterations to fatty acid metabolism also alter sleep parameters. For example, ACADS, a component of the β-oxidation pathway, has been shown to affect rapid eye movement sleep, as its deficiency affects theta oscillations during sleep (133).

Fatty acid metabolism is also disrupted by high fat diets in drosophila (134), which may therefore have a downstream consequence on sleep. Interestingly, it has been found that medium- and long-chain dietary fatty acids, which range from approximately 6-10 carbons in length, promote sleep in flies (135), although their varying effects on sleep duration, number of sleep bouts, and activity during wakefulness vary massively. This may be as a result of differential oxidation of fatty acid species (136), as free fatty acids can be either used to produce Acetyl-CoA or stored as triglycerides, which may affect their relative effects on sleep architecture.

## 4. Clinical evidence in humans

The composition of the diet can influence both sleep quality and structure (137–146). Anyway, in humans, establishing the effects of a specific nutrient on sleep parameters is very complex, because they are not consumed isolated but within dietary patterns. A recent interesting review summarized many findings regarding the effects of macronutrients on sleep in humans (147).

Some studies observed that a healthy dietary pattern, with a large consumption of fruit, vegetables, fish and fibers, and regular meals, is associated to a better sleep quality (139, 148–150). The consumption of specific variety of cherries or their juice, which are rich in melatonin and serotonin, is often reported to be associated to a better sleep (151, 152). Vegetarians seem to have less sleep disorder, quantified with a lower Athens Insomnia Scale score (given by the sum of the scores for difficulty in sleep induction, awakenings during the night, early-morning awakening, total sleep time, overall quality of sleep and day functioning) and lower diurnal sleepiness, than non-vegetarians, and this is particularly evident in women (153). Anyway, the unprocessed red meat is not clearly related to a better or a poorer sleep (148). The Mediterranean diet, which is based on vegetables, fruit, whole grain and fish consumption, is associated with a better sleep quality, in particular with adequate sleep duration, reduced sleep latency, better sleep efficiency and reduced day dysfunction (154, 155).

Some studies reported that protein intake is associated to a better sleep quality and duration (142, 144–146, 156) but not all studies are completely concordant (138, 144, 145).

As already discussed, Trp is an essential amino acid that, after passing the brain blood barrier, is converted in serotonin and ultimately to melatonin, which promotes sleep. Sleep duration is positively associated with the dietary Trp: LNAA ratio (37). The reason for which this ratio among dietary intake of Trp and LNAA is related to sleep duration may be due to the fact that LNAA compete with Trp to bind to a carrier protein to pass the blood–brain barrier (157), influencing brain Trp bioavailability.

A study in more than 1,900 Japanese healthy adults observed an association between self-reported quality of sleep and self-assessed dietary habits. In terms of food, the intake of pulses, bread, fish and shellfish was correlated with sleep duration. Interestingly, these associations were present only in men, and not in women. The authors observed that sleep duration was directly related to the protein intake (140). A study in 104 healthy adults and older individuals from Singapore investigated the relationship among protein intake and sleep, focusing on tryptophan intake (37). Sleep duration was positively associated to dietary Trp: LNAA ratio but also to plant Trp and plant Trp: LNAA, and this interestingly suggest that the source of protein may play a role. Plant sources had a higher Trp:LNNA ratio, in comparison with animal sources, and moreover, they contain carbohydrates, which can increase plasma Trp:LNAA up to 50%. Dairy protein intake was negatively associated with sleep duration, despite its high content of tryptophan. Also sleep efficiency seemed to be negatively influenced by protein from diary sources, since people with a sleep efficiency of more than 85% ate less protein from diary sources in comparison with people with lower sleep efficiency (*p* = 0.033). This result may be related to the fact the dairy products contain not just alpha-lactalbumin, but also other proteins, which may increase the LNAA amount and therefore reduce the ratio. The different effect of the protein quality on sleep may also be related to the interaction of the gut microbiota with the peptide produced with the protein digestion, with the eventual development of dysbiosis and inflammation (150), but not all studies observed this different effect on sleep by proteins of different sources (146). Similarly, the ingestion of an evening whey protein supplementation, rich in tryptophan, did not improve sleep parameters in 15 elite male athletes. However, as authors point out, the high protein/energy intake snack did not negatively affect sleep, which is interesting for the importance of this kind of snacks for athlete recovery (158). Another study in 36 elite male football players observed that evening protein intake was associated with shortened sleep onset latency (159).

Tryptophan supplementation from non-diary sources is not invariably associated to an amelioration of sleep quality. In a recent study, 22 women with fibromyalgia were administered an eucaloric Mediterranean diet enriched with walnuts as source of tryptophan and magnesium for 16 weeks, but sleep quality did not ameliorate (160).

Data on carbohydrate intake and sleep are controversial. High- CHO (carbohydrate) diets may reduce the sleep onset latency (161). The glycaemic index may influence this parameter, as a study observed that the consumption of a high glycaemic index meal 4 h before bedtime shortened the sleep latency (113). Other sleep parameters were not affected. High-glycaemic index carbohydrates can increase the ratio tryptophan: LNAA through insulin action, which promotes the uptake of LNAA from muscles (162). Furthermore, the timing of carbohydrate intake plays a role, as the same high glycaemic index meal given 1 h before bedtime did not have a significant effect on sleep latency (113). On the other hand, another study reported no changes in sleep onset nor sleep efficiency, but a reduction in the restorative sleep in the first sleep cycle, after consumption of a high CHO meal 4 h before bedtime (163), and another one reported a reduced sleep quality associated to a high CHO intake (139). In fact, the consumption of higher percentage of energy from carbohydrates has been associated with arousals (137). A study in 32 female athletes reported an increase in wake after sleep onset and decrease of sleep efficiency along with increase in CHO intake, and on the contrary a decrease of sleep onset latency along with saturated fat intake (164).

In healthy adults administered a very low carbohydrate diet, the slow wave phase increased, and the rapid eye movement (REM) sleep decreased, in comparison to an isocaloric control mixed diet (165). Another study observed a reduction of sleepiness during a very low-calorie ketogenic diet during the reduced ketosis phase, but no evidence of modifications of other sleep parameters (166).

Regarding fat intake, less data is available. A short sleep is often associated to a higher fat intake (143), but this is not invariably observed (167). Conversely, saturated fats, which are commonly present in the Western diet, have a negative effect on sleep quality according to some studies. *Ad libitum* eating over 3 days, with a great intake of saturated fat and sugars, is associated to a less restorative sleep and increased latency (137). In particular, the percent of energy from saturated fat predicted a reduced SWS. On the other hand, other studies reported a better self-reported sleep with a high-fat diet (168), or a neutral effect (161, 163).

The effects on sleep of omega-3, of which fatty fishes are rich, and omega-6 long-chain polyunsaturated fatty acids, are not completely elucidated yet (169, 170). Fish consumption has been often associated to a better sleep quality evaluated with the Pittsburgh Sleep Quality Index Questionnaire and a longer sleep duration in men (139, 140).

As some interesting reviews and metanalysis recently pointed out, at the moment there are no striking evidence about the effects of a specific nutrient on sleep, and further research is warranted (150, 156, 171).

Conversely, deprivation of sleep can induce a modification in the pattern of the preferred foods. In fact, short sleepers, very short sleepers and individuals after a nightshift tend to eat less healthy food, increase their intake of saturated fats and carbohydrates, and consume less vegetables (20, 22). The mechanisms through which this happens may be related to a longer time of food availability, or research for gratification through calorie-dense food, or to higher energy needs to maintain the extended wakefulness, or to imbalances in appetite hormones (172).

The time of the administration of food may influence sleep and circadian rhythm. Time-restricted eating (TRE) consists in eating within a restricted time window during the day, and fast for the rest of the day. TRE is used mainly in obesity, since it favors weight loss and an amelioration of metabolic disturbances (173). Since time-restricted feeding (TRF) has been linked with an alignment of circadian rhythms in animal models, and the prolonged fasting stimulates SIRT-1 activity and ketone bodies production, which can influence circadian rhythm, it has been hypothesized that TRE may influence sleep. A recent review of human trials studied the effects of TRE on sleep in overweight and obese humans, trough questionnaires, self-reported diaries, and accelerometers (174). The authors reported that overall sleep quality did not change, sleep duration did not increase, nor insomnia was modified. Of note, the effects on sleep latency and efficiency did not align across studies. The insignificant impact on sleep quality may be attributed to the minimal weight loss occurred; the duration of sleep as well as the presence of insomnia remained unchanged probably due to the fact that participants in the trials were already sleeping for more than 7 h per night and did not suffer from insomnia. Therefore, TRE deserves more studies on larger samples of patients.

Some minerals and vitamins have been related to sleep duration and efficiency, in particular sodium, iron, zinc and vitamins D, B9, and B12 (37, 140, 164), but this topic goes beyond the aim of our review.

## 5. Conclusion

Dietary composition and sleep are bidirectionally related. Macronutrients influences sleep, and on the other hand, lack of sleep is related to the preferential choice of certain food. The mechanisms through which this mutual influence takes place, remain poorly defined. Both sleep processes C and S are involved. The role of circadian clocks in metabolic homoeostasis and the implications for health and disease are gradually being elucidated. However, besides regulating the metabolism of macronutrients, conversely the circadian clock is also regulated by macronutrients, both centrally and peripherally. Furthermore, regulation of peripheral circadian clocks can feed back on to the central pacemaker in the SCN.

Clinical studies have been inconclusive regarding the effects of a single macronutrient on sleep parameters in humans, probably also because the inevitable association of the macronutrients in dietary patterns.

The effects of food on sleep are not connected just to the food itself, but comprehend the response that its taste and its smell elicit in the organism. In fact, sweet taste is able to modify sleep and reduces sleep deprivation due to starvation, and the odors are able to influence sleep latency, duration and quality, and to induce a metabolic effect in animal models. These data may be of interest for ameliorating the management of patients suffering from sleep deprivation. If these results are translatable in humans, it has still to be determined.

Further research should be conducted to explore TRE as a tool for managing sleep disruption in humans: in obese patients, where the effect may be related to the weight loss achieved, but also in normal weight individuals suffering from sleep disturbances.

Further studies aimed to better understand the relationship between nutrients and sleep are needed, given the importance of sleep for metabolic health in humans.

## Author contributions

EG: conceptualization. EG, MB, and MV: writing and original draft preparation. AL, LG, and DR: review and editing. All authors contributed to the article and approved the submitted version.

## Funding

This research was funded by PRIN 2020 (grant no. 2020NCKXBR), Italian Ministry of Education, Universities and Research; co-funding of the European Union - Next Generation EU, Mission 4 Component 2 Investment 1.5, project Rome Technopole - code ECS 00000024 (CUP: B83C22002820006).

## Conflict of interest

The authors declare that the research was conducted in the absence of any commercial or financial relationships that could be construed as a potential conflict of interest.

## Publisher’s note

All claims expressed in this article are solely those of the authors and do not necessarily represent those of their affiliated organizations, or those of the publisher, the editors and the reviewers. Any product that may be evaluated in this article, or claim that may be made by its manufacturer, is not guaranteed or endorsed by the publisher.
